# Need for follow-up studies to better understand the relationship between the rs7041 variant and pancreatitis in COVID-19 patients

**DOI:** 10.1590/1806-9282.20250610

**Published:** 2025-08-08

**Authors:** Zhe-Ying Jiang, Wei-Jia Meng, Cui-Ping Li, Lian-Ping He, Hua-Qin Su

**Affiliations:** 1 Taizhou University, School of Medicine – Taizhou, China.

Dear Editor,

We are pleased with the study by Öztürk et al.^1^, which demonstrated that patients with coronavirus disease 2019 (COVID-19) carrying the rs7041 variant in the vitamin D-binding protein (VDBP) gene may have an increased susceptibility to pancreatitis. However, I think there are still some issues that need to be addressed.

The primary issue of this study is that the increased susceptibility to pancreatitis in patients infected with the novel coronavirus may not be solely attributed to the rs7041 gene variant. The study conducted by Öztürk et al.^1^ aimed to investigate the association between the rs7041 variant of the VDBP gene and the susceptibility to pancreatitis in patients with COVID-19. It is insufficient to examine the susceptibility of COVID-19 patients to pancreatitis solely based on variations at the rs7041 locus. A comprehensive analysis should also consider additional genes and genetic factors, as well as lifestyle influences, that may contribute to pancreatic involvement in patients with COVID-19.

In fact, genetic variation at the rs7041 locus alone does not necessarily confer an increased susceptibility to pancreatitis in patients with COVID-19. The theoretical basis of the study by Öztürk et al.^1^ is that the rs7041 variation affects the binding ability of VDBP to vitamin D and its normal function in the body. However, the metabolism of vitamin D plays a decisive role in many diseases^2-5^. When the metabolism and function of vitamin D are compromised, normal physiological processes such as immune regulation, cell growth, and differentiation are also disrupted, thereby increasing pancreatic cells’ sensitivity to damage and consequently raising the risk of pancreatitis. The rs7041 is not the only gene that influences the metabolism and function of vitamin D. The rs4588 variant in VDBP has been shown to affect vitamin D levels, suggesting that this variant may also impact VDBP's binding capacity to vitamin D and its normal physiological functions. Individuals carrying the rs4588 CC genotype exhibit significantly higher serum total 25-hydroxyvitamin D [25(OH)D] levels compared to those with the AA genotype, indicating that this polymorphism plays a role in vitamin D metabolism^6^.

Furthermore, studies have demonstrated that variations in the ACE gene may elevate the incidence of pancreatic-related diseases in patients with COVID-19. Specifically, the insertion/deletion polymorphism of the ACE gene has been shown to correlate with changes in enzyme activity, which in turn heightens the risk of hypertension and associated complications, including pancreatic diseases^7,8^.

Meanwhile, the mean age of the pancreatic involvement patient group (64.73 years) and the control group (57.55 years) shows a certain difference. Studies have demonstrated that age is closely associated with genetic mutations, with the likelihood of such mutations increasing as individuals age^9^. Consequently, the age factor may contribute to a higher frequency of the rs7041 variant in the pancreatic involvement patient group, potentially confounding the study results.

The incidence of pancreatitis is closely related to lifestyle such as diet^10^, smoking status^11,12^, and alcohol consumption^13^, which could influence both the development of pancreatitis and the body's vitamin D metabolism. However, no lifestyle-related data were collected in this study. These factors potentially influence the relationship between the VDBP gene rs7041 variant and pancreatic involvement in COVID-19 patients.

Overall, while this study offers valuable insights into the potential association between the VDBP gene rs7041 variant and pancreatic involvement in COVID-19 patients, several aspects require improvement to enhance the robustness and generalizability of the results.

## Data Availability

The datasets generated and/or analyzed during the current study are available from the corresponding author upon reasonable request.
